# Identifying Double Bond Positions in Phospholipids Using Liquid Chromatography-Triple Quadrupole Tandem Mass Spectrometry Based on Oxygen Attachment Dissociation

**DOI:** 10.5702/massspectrometry.S0080

**Published:** 2020-01-22

**Authors:** Hidenori Takahashi, Yuji Shimabukuro, Daiki Asakawa, Akihito Korenaga, Masaki Yamada, Shinichi Iwamoto, Motoi Wada, Koichi Tanaka

**Affiliations:** 1 Shimadzu Corporation, 1 Nishinokyo-Kuwabara-cho, Nakagyo-ku, Kyoto 604–8511, Japan; 2 Graduate School of Science and Engineering, Doshisha University, 1–3 Kyotanabe, Kyoto 610–0321, Japan; 3 National Institute of Advanced Industrial Science and Technology (AIST), National Metrology Institute of Japan (NMIJ), Tsukuba Central 2, 1–1–1 Umezono, Tsukuba, Ibaraki 305–8568, Japan

**Keywords:** radical-induced dissociation, hydroxyl radical, atomic oxygen, collision cell, hydrogen attachment/abstraction dissociation

## Abstract

Lipids, a class of biomolecules, play a significant role in the physiological system. In this study, gas-phase hydroxyl radicals (OH·) and atomic oxygens (O) were introduced into the collision cell of a triple quadruple mass spectrometer (TQ-MS) to determine the positions of the double bond in unsaturated phospholipids. A microwave-driven compact plasma generator was used as the OH·/O source. The reaction between OH·/O and the precursor ions passing through the collision cell generates product ions that correspond to the double bond positions in the fatty acyl chain. This double bond position specific fragmentation process initiated by the attachment of OH·/O to the double bond of a fatty acyl chain is a characteristic of oxygen attachment dissociation (OAD). A TQ-MS incorporating OAD, in combination with liquid chromatography, permitted a high throughput analysis of the double bond positions in complex biomolecules. It is important to know the precise position of double bonds in lipids, since these molecules can have widely different functionalities based on the position of the double bonds. The assignment of double bond positions in a mixture of eight standard samples of phosphatidylcholines (phospholipids with choline head groups) with multiple saturated fatty acyl chains attached was successfully demonstrated.

## INTRODUCTION

Lipids are a structurally diverse class of molecules that can be differentiated based on their distinct head groups, alkyl chains, the position of the double bond, and geometries such as stereospecific numbering (*sn*), degree of *cis*–*trans* isomerization, and other alkyl chain modifications. Lipids with the same molecular mass may have completely different biological functions, depending on the position of the double bond in the fatty acyl chain.^1)^ While conventional collision induced dissociation (CID) based liquid chromatography-tandem mass spectrometry (LC-MS/MS) is an effective analytical technique for characterizing the head group, alkyl chain length, and stereospecific numbering,^2,3)^ identifying the position of the double bond in complex lipid samples containing numerous unsaturated species is still a difficult task. To address the limitations of conventional LC-MS/MS in the structural analysis of lipids, several complementary techniques have been proposed.^4–6)^ In 2018, we developed a novel radical-induced MS/MS technique called oxygen attachment dissociation (OAD)-MS/MS, which allows double bond-specific dissociation upon the attachment of oxygen.^7)^ OAD-MS/MS utilizes the interaction between the precursor ion and the neutral radical species of hydroxyl radical (OH·) and/or atomic oxygen (O) generated by the microwave discharge of water vapor and enables the position of the double bond to be assigned. Methylene bridges adjacent to the double bond are selectively fragmented by OH· and/or O upon the oxidation of the double bond. These fragmented ions are useful in terms of understanding the detailed structure of lipids in complex biological matrices. In contrast to other similar double bond-specific fragmentation techniques such as ozone-induced dissociation (OzID),^5)^ OAD-MS/MS is more laboratory-safe and operationally simple, because ozone, a strong oxidant, can be hazardous, even at low concentrations. Additionally, the fragmentation efficiency of OAD-MS/MS can be higher than that of OzID under high vacuum (< 0.1 Pa). In fact, an ion mobility cell filled with a high ozone pressure (≈10 Pa) is used for OzID in conventional LC-MS/MS for realizing high throughput analysis.^8)^ In contrast, OAD-MS/MS can be coupled to a conventional collision cell of a triple quadruple mass spectrometer (TQ-MS) under a high vacuum (< 0.1 Pa), which significantly reduces the price and complexity of the equipment. In our previous study, OAD-MS/MS was demonstrated using matrix-assisted laser desorption/ionization (MALDI) ion trap time-of-flight mass spectrometry (IT-TOF MS).^7)^ However, the relatively long reaction time (> 100 ms) required the fragmented ions that need to be detected made the coupling to the conventional LC-TQ-MS system inefficient. To couple OAD-MS/MS to the conventional LC-TQ-MS system, the O/OH density inside the reaction cell should be enhanced by around ten-fold, because the flight time of ions passing through the collision cell is substantially less than 10 ms. To achieve this, we applied a microwave-driven *inductively* coupled plasma (ICP) radical source^9)^ to OAD-MS/MS in this study. In our previous study, a microwave-driven *capacitively* coupled plasma (CCP) radical source was utilized for OAD-MS/MS.^7)^ As reported by Shimabukuro and co-workers, the microwave-driven ICP source generates a higher radical flux than the microwave-driven CCP source at a high vacuum (< 0.1 Pa).^10,11)^ Since the gas pressure of the collision cell is equivalent to the operating pressure of the microwave-driven ICP (≈ 0.1 Pa), the latter can be directly connected to the collision cell without the need for differential pumping. This significantly improves the transport efficiency of the generated radical species into the collision cell. Herein, we coupled LC-TQ-MS and OAD-MS/MS (using ICP) and utilized this system for the determination of the position of double bond in unsaturated phospholipids.

## EXPERIMENTS

### Materials

Standard phospholipids (Table 1) were purchased from Avanti Polar Lipids (Alabaster, AL). Each sample was dissolved in methanol, and the solution was diluted to the final concentration of 1 μM in 80% (20 mM ammonium formate)/20% (acetonitrile (ACN))/isopropanol (IPA) mixture, 1/1, v/v) solution. Each diluted solution was mixed with an equal volume into a single tube.

**Table 1:** Table 1. Standard phospholipids used in this study.

Lipid name	Exact mass
PC (18 : 0/18 : 0)	789.625
PC (18 : 1(9*Z*)/16 : 0)	759.578
PC (18 : 0/18 : 1(9*Z*))	787.609
PC (14 : 1(9*Z*)/14 : 1(9*Z*))	673.468
PC (16 : 1(9*E*)/16 : 1(9*E*))	729.531
PC (18 : 1(6*Z*)/18 : 1(6*Z*))	785.593
PC (18 : 3(9*Z*,12*Z*,15*Z*)/18 : 3(9*Z*,12*Z*,15*Z*))	777.531
PC (16 : 0/20 : 4(5*Z*,8*Z*,11*Z*,14*Z*))	781.562

### Liquid chromatography

A Shimadzu Nexera LC system was used for the separation of the lipid mixture. Briefly, a 5 μL aliquot of a solution of the lipid mixture was injected using an autosampler (SIL-30AC; Shimadzu, Japan) into a core-shell column (Phenomenex Kinetex™ 2.6 μm, C8 100 Å, 50×2.1 mm column) at a flow rate of 0.5 mL/min. The samples were eluted by step gradient of mobile phase A (20 mM ammonium formate aqueous solution) and mobile phase B (ACN/IPA mixture, 1/1, v/v). The elution was carried out as follows: 0 to 1 min, 20% B; 1 to 2 min, a linear gradient from 20 to 40% B; 2 to 25 min, a nonlinear (exponential) gradient from 40 to 92.5% B; 25 to 26 min, a linear gradient from 92.5 to 100% B; 26 to 35 min, 100% B to wash the column; 35 to 38 min, 20% B to re-equilibrate.

### Tandem mass spectrometer and ICP source

**Figure 1:**
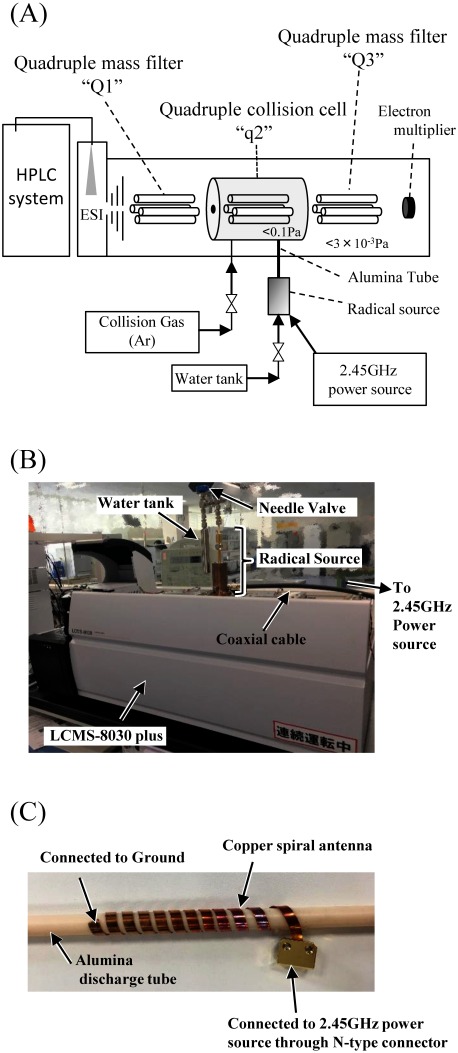
Fig. 1. (A) Schematic diagram of triple quadruple mass spectrometer modified for OAD-MS/MS. (B) Picture of OAD-MS/MS system. (C) Picture of the alumina discharge tube of radical source.

All MS and MS/MS experiments were performed using a TQ-MS equipped with an electrospray ionization (ESI) source (LCMS-8030 plus, Shimadzu Corporation, Japan), as shown in Fig. 1. An alumina tube (i.d. 4 mm) was connected to the collision cell to introduce OH· and O generated by the radical source based upon a microwave-driven ICP.^11)^ A 0.3-mm-thick, 3.0-mm-wide copper ribbon wound around a 6 mm outer diameter 4 mm inner diameter alumina discharge tube (Nikkato Corp., Japan) served as the antenna for the 2.45 GHz power source (Tokyo Keiki Inc., TMEB101B00B) delivered *via* an N-type connector as shown in Fig. 1(C). A cylindrical Nd–Fe magnets (φ26.9 mm×φ14 mm×64 mm) forms a magnetic field structure inside the alumina discharge tube with the region of the intensity greater than 875 G corresponding to an electron cyclotron resonance (ECR) condition at 2.45 GHz in the axial direction. Water vapor was introduced into the discharge tube at a flow rate controlled by a needle valve (US-916P-P6.35, Fujikin, Japan) below 1 sccm. Ultra-pure water (Milli-Q, Japan Millipore Co., Ltd., Tokyo) stored in a reservoir tank reached the inlet of the needle valve at room temperature. High quality H_2_^18^O (98 atom%), which was used in some specific experiments was purchased from Taiyo Nissan Co., Ltd. The microwave power below 10 W was applied to the copper spiral antenna, and consequently, the microwave discharge of water vapor generated OH· and O. The ICP source does not expose any metallic parts from the water vapor contributing to reduce recombination of OH· and O at the metallic wall.

The gas pressures inside and outside the collision cell was maintained below 0.1 and 3×10^−3^ Pa, respectively, which were within the normal range for TQ-MS. The presence of radical species inside the radical source was confirmed by optical emission spectroscopy using a compact optical spectrometer (Ocean optics USB 2000+ and Ocean optics Flame-S).

## RESULTS AND DISCUSSION

### Microwave discharge of H_2_O

**Figure 2:**
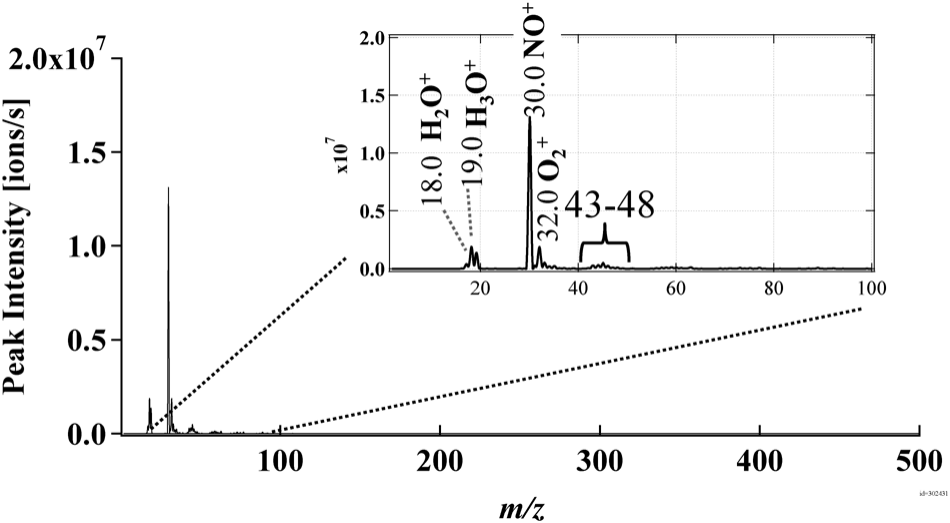
Fig. 2. MS spectrum obtained by Q3 scan in the microwave discharge of water, without injecting the sample.

We first analyzed the composition of the ions and neutral reactive species generated by the microwave discharge of water. For this, the third quadrupole mass filter (Q3) was scanned from *m*/*z* 2 to 500 in the microwave discharge of water, without injecting the sample. Figure 2 shows the mass spectrum obtained for a Q3 scan at a scan speed of 1,000 unit/s. Since no ions were observed when the microwave discharge was turned off (data not shown), it was concluded that the observed ions originated from the products of the microwave discharge. Abundant H_2_O^+^, H_3_O^+^, NO^+^, and O_2_^+^ peaks were observed at *m*/*z* 18.0, 19.0, 30.0, and 32.0, respectively. Ions observed between *m*/*z* 43 and 48 can be attributed to minor contaminants. Species such as NO^+^ and the minor contaminants originate because of the microwave discharge of residual background gas (*i.e.*, air and impurities) inside the radical source. To avoid introducing these ions into the collision cell, ion deflection magnets can be placed at the exit of the radical source. In this study, the target precursor ions for OAD-MS/MS are only singly charged positive ions. Therefore, the population of the observed ions would be negligible in OAD-MS/MS because of the Coulombic repulsion among them.

**Figure 3:**
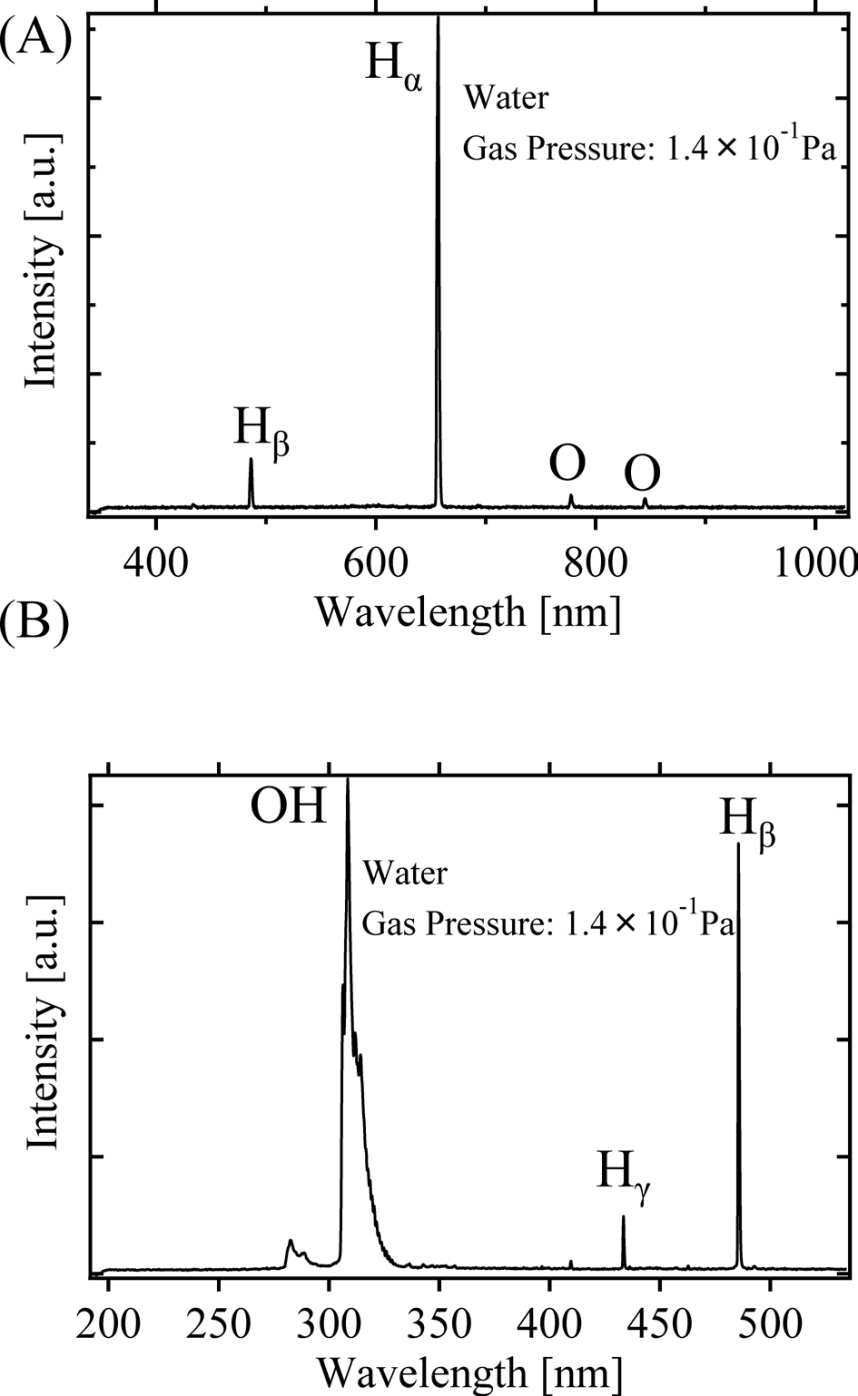
Fig. 3. Optical emission spectra of microwave water ICP at 1.4×10^−1^ Pa, 10 W: (A) visible light region and (B) ultraviolet region.

Figure 3 shows the optical emission spectrum of the microwave discharge of water. The optical emission spectrum gives a rough estimate of the neutral reactive species generated by the discharge. The optical emission spectroscopy was performed in a separate experimental setup, that had been disconnected from the mass spectrometer. The microwave discharge of water contained H·, O, and OH·. The emission signal from OH· was observed in the ultraviolet region at 309 nm (A^2^Σ^+^-X^2^Π),^12)^ while that from O was observed at 845 nm.^13)^ In addition, the microwave discharge resembles the atomic hydrogen spectrum up to the Balmer gamma and delta lines (410 and 434 nm), which correspond to the higher excited levels of hydrogen atoms. Since the generated radicals were transported from the radical source to the collision cell *via* the alumina tube (Fig. 1), the radicals are cooled to room temperature owing to the collision with the inner surface of the alumina tube. These low-temperature OH· and O species can initiate the OAD, as reported in our previous study.^7)^ Low-temperature H· radicals, on the other hand, do not play any significant role in the dissociation of double bonds.^7)^

### OAD-MS/MS of phospholipid using TQ-MS

**Figure 4:**
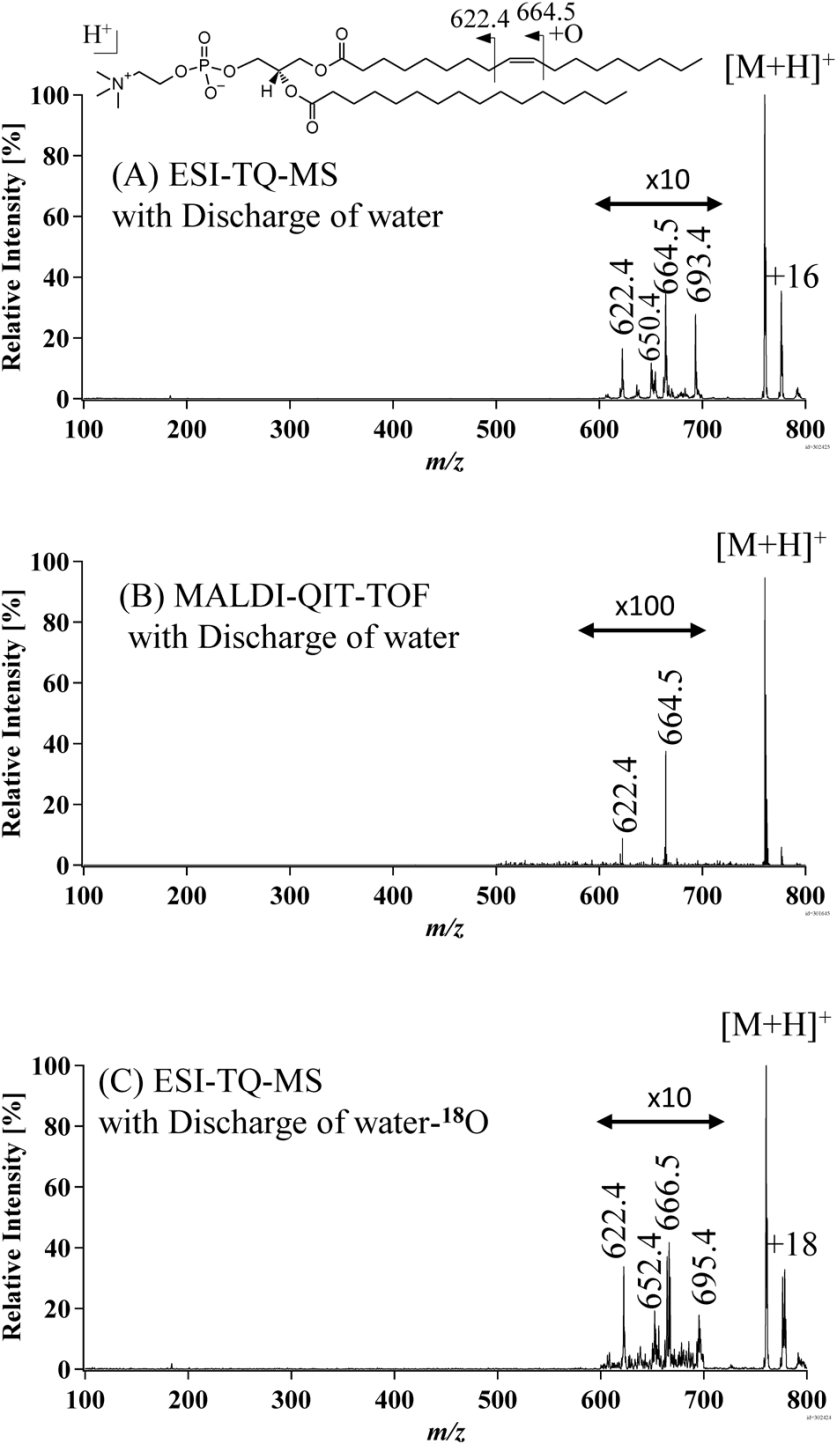
Fig. 4. OAD-MS/MS spectrum of PC(18 : 1/16 : 0) with microwave discharge of water using (A) ESI-TQ-MS and (B) MALDI-QIT-TOF. (C) OAD-MS/MS spectrum with microwave discharge of H_2_^18^O using ESI-TQ-MS.

To demonstrate the utility of OAD-MS/MS for the analysis of phospholipids, PC 18 : 1/16 : 0 was used as a model and was infused by syringe pump (0.5 mL/min) and ionized by ESI (3.5 kV capillary voltage). The precursor ion was isolated in Q1 and fragmented in the collision cell (q2) containing OH·/O. The product ions were scanned in Q3. The scan speed of Q3 was set to be 1,000 unit/s. Figure 4(A) shows the OAD-MS/MS spectrum [M+H]^+^ of the model phospholipid, PC 18 : 1/16 : 0. As in the case of previously reported MALDI-QIT-TOF based OAD-MS/MS result,^7)^ OAD performed by TQ-MS/MS provided abundant fragment ions at *m*/*z* 622.4, 650.4, 664.5, and 693.4, which are formed by the oxidation of the C=C double bond accompanied by C–C bond cleavage adjacent to the oxidation site. The proposed structures of the fragmented ions are shown in Scheme 1. As a result, the present TQ-MS/MS based method is useful for the identification of double bond position in a lipid acyl chain.

**Figure:**
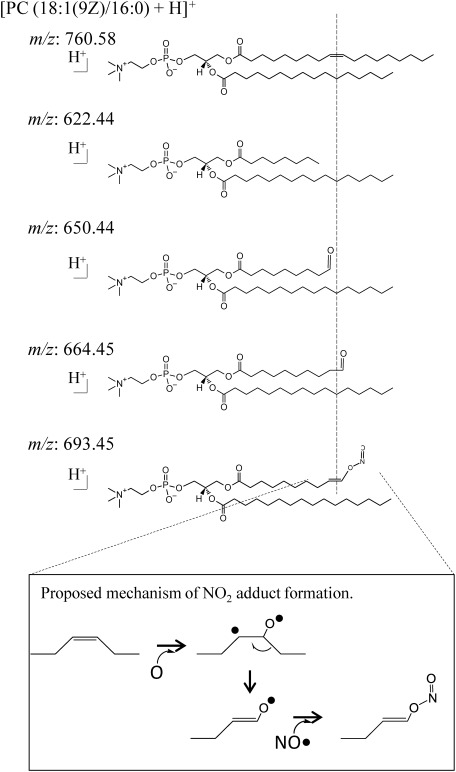
Scheme 1. Proposed structure of fragment ions observed in Fig. 4.

We next focused on the fragment ion at *m*/*z* 693.4, which is observed as an odd nominal mass, although other fragments also appear as even nominal masses. To simplify the peak assignment, we applied the so-called nitrogen rule, in which a peak pair with odd and even nominal masses (in this case, a peak pair of ions at *m*/*z* 664.5 and 693.4) are considered. According to this rule, the ion at *m*/*z* 693.4 can be considered to be formed by the oxidative C–C bond cleavage with a subsequent NO· attachment (Scheme 1). Unlike the case of O and OH·, NO· does not become attached to a C=C double bond. In contrast, NO· can become attached to the alkoxy radical, which is considered to be the intermediate for the oxidative C–C bond cleavage and the corresponding reaction rate was reported to be 4.4×10^−11^ cm^3^/molecule^−1^s^−1^.^14)^ As a consequence, we conclude that the ion at *m*/*z* 693.4 contains an –ONO group, as shown in Scheme 1. For comparison, OAD-MS/MS was performed using the same radical source in the MALDI-QIT-TOF MS used in our previous study^7)^ (Fig. 4(B)). However, the peak at *m*/*z* 693.4 for the nitrogen adduct was absent in this experiment, indicating that the background nitrogen gas was contributed from the atmospheric ESI source and/or solvent.

To confirm the proposed structures of the fragmented ions (Scheme 1), the microwave discharge of H_2_^18^O was employed instead of H_2_^16^O for the OAD-MS/MS (Fig. 4(C)). While the peak at *m*/*z* 622.4 was unchanged, peaks at *m*/*z* 650.4, 664.5, and 693.4 were increased by 2.0 Da, indicating that these peaks correspond to the oxidized form, wherein ^16^O is replaced by ^18^O. This result supports the proposed structure of the fragmented ions shown in Scheme 1. Interestingly, the ONO-adduct ion observed at *m*/*z* 693.4 is increased by only 2.0 Da. If both the oxygen atoms of ONO were to have been replaced by ^18^O, the mass increase should have been 4.0 Da. Therefore, a *m*/*z* 693.4 product ion would be induced by the attachment of NO· to the oxidized C=C specific fragment of *m*/*z* 664.5. Since NO· is generated by the microwave discharge of the residual gas from atmospheric ESI source, and not from H_2_^18^O, the mass of the –ONO adduct ion observed at *m*/*z* 693.4 would be increased only by 2.0 Da.

**Figure 5:**
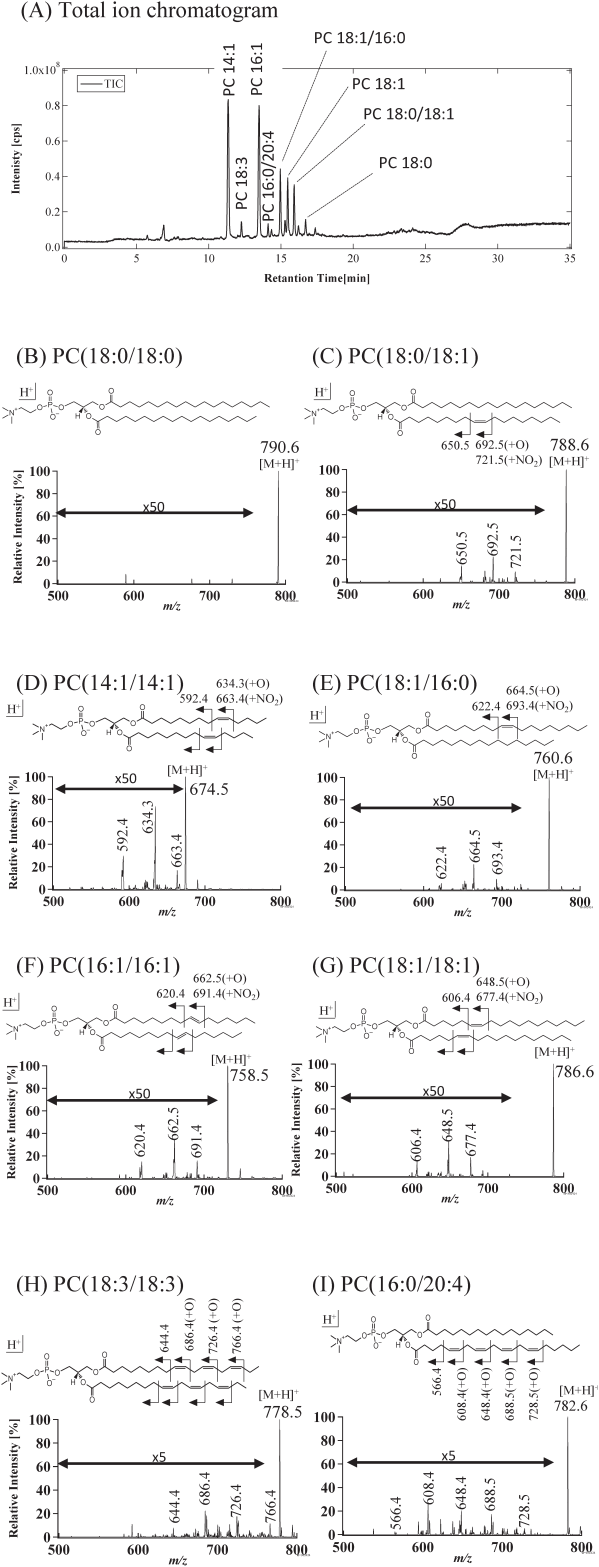
Fig. 5. (A) TIC of phospholipid mixture and (B)–(I) OAD-MS/MS spectra of each phospholipid obtained by one single product ion scan with 0.5 s.

We next utilized OAD-MS/MS for the LC-MS of the lipid mixture listed in Table 1. The auto-MS/MS mode (data dependent acquisition) with a cycle time of 1 s was used for this purpose. MS was performed in the Q3 scan mode with a scan time of 0.5 s. Following the MS experiment, the ion with the highest intensity (> 1×10^6^ cps (counts per second)) was selected and simultaneously analyzed by OAD-MS/MS from *m*/*z* 500–1,000, with a scan time of 0.5 s. Figure 5(A) shows the total ion chromatogram (TIC, *m*/*z* 500–1,000). Each standard phospholipid injected was clearly separated by the LC. Figures 5(B)–(J) shows OAD-MS/MS spectrum of each phospholipid obtained by single scan with a scan time of 0.5 s. While no product ions were observed for the saturated phospholipid of PC (18 : 0/18 : 0) (Fig. 5 (B)), abundant diagnostic product ions were observed for all the unsaturated phospholipids (Figs. 5 (C)–(I)). As observed in the OAD-MS/MS experiment using a syringe pump (Fig. 3), triplet peaks (non-oxidized, oxidized, and nitric-oxidized ions) appear at around the double bond corresponding to the position of the double bond. Although the OAD-MS/MS spectrum of phospholipid with multiple saturated fatty acyl chain (Figs. 5 (H) and (I)) becomes complex owing to overlapping multiple triplet peaks, the spectrum can be interpreted by analyzing each triplet peak. As shown in Fig. 4(B), the accuracy of the peak assignment can be further enhanced by comparing the results of the microwave discharge of H_2_^16^O and H_2_^18^O.

## CONCLUSION

Phospholipids were analyzed by OAD-MS/TQ-MS coupled with LC, in order to identify the double bond positions in standard phospholipids. The ICP radical source generated OH· and O with higher fluxes and lower operational pressure compared to the previously developed CCP radical source. The new ICP radical source permitted a successful direct connection to the conventional collision cell of TQ-MS to be realized without the need for a differential pumping system. Similar to the findings reported in our previous study on the use of MALDI-IT-TOF-MS in OAD-MS/MS,^7)^ the methylene bridges adjacent to the double bonds were selectively oxidized and subsequently fragmented by OH· and O. These fragmented ions can be used for the high throughput analysis of the double bond positions in the collision cell. OAD-MS/MS, in combination with LC, is a promising new analytical technique for understanding the detailed structure of lipids in complex biological matrices.

## Notes

Mass Spectrom (Tokyo) 2019; 8(2): S0080
